# Descriptive analysis of adverse events following immunization with oral cholera vaccine in Lebanon

**DOI:** 10.3389/fpubh.2024.1480744

**Published:** 2024-11-20

**Authors:** Abeer Zeitoun, Aya Ibrahim, Sarah Reda El Sayed, Eva Hobeika, Rita Karam

**Affiliations:** ^1^Quality Assurance of Pharmaceutical Products Department, National Pharmacovigilance Program, Lebanese Ministry of Public Health, Beirut, Lebanon; ^2^Faculty of Arts and Sciences, Holy Spirit University of Kaslik, Beirut, Lebanon; ^3^Department of Chemistry and Biochemistry, Faculty of Sciences, Lebanese University, Beirut, Lebanon; ^4^Pharmacology Department, Faculty of Medical Sciences, Lebanese University, Beirut, Lebanon

**Keywords:** oral cholera vaccine, adverse events following immunization, vaccines, pharmacovigilance, cholera, descriptive analysis

## Abstract

**Background:**

A national Oral Cholera Vaccine (OCV) Euvichol-Plus® campaign was launched in Lebanon, in response to the first outbreak in three decades, recorded in October 2022. The OCV vaccination campaign was carried out between November 2022 and February 2023. This study aims to cover adverse events reports, received at the Lebanese National Pharmacovigilance Program’s (LNPVP) passive surveillance system.

**Methods:**

Case reports were extracted from the LNPVP’s database. SPSS software was used to perform statistical analysis, with categorical variables compared using Pearson’s *χ*^2^ test. A descriptive analysis was performed based on age, gender, vaccine administered, and adverse event(s) associated with the administered vaccine.

**Results:**

A total of 115 Adverse Events Following Immunization (AEFIs) were reported, which corresponded to 46 case reports. The top three reported AEFIs were fever (39.13%), diarrhea (30.43%), and vomiting (30.43%). Reported cases were non-serious (82.6%). The highest proportion of Individual Case Safety Reports (ICSRs) received is attributed to females (56.5%), and the age category of 2 and 11 years old (41.3%). Reporters’ age range was 1–74 years old.

**Conclusion:**

Monitoring AEFIs through the cholera outbreak’s emergency campaign favors the safety profile of OCV.

## 1 Introduction

Cholera is an intestinal bacterial infection caused by *Vibrio cholerae*. It is transmitted by several means including person-to-person contact, contaminated water, and contaminated food. It is characterized by a short incubation period between 2 hours and 5 days. The disease is marked by hallmark secretory diarrhea, yet most patients are asymptomatic. Only less than 20% of patients develop acute watery diarrhea with severe dehydration exposing them to rapid loss of body fluids and even death if left untreated (1). The infection can be confirmed by accurate and accessible techniques such as Polymerase Chain Reaction (PCR) and stool Rapid Diagnostic Tests. Despite being easily treatable, with rapid rehydration being the primary treatment, either through Oral Rehydration Therapy (ORT), or intravenous fluids in severe cases, cholera continues to be a global public health threat occurring as an endemic disease in some regions and is causing major epidemics in some Low- and Middle-Income Countries (LMICs) (1).

Cholera is an infection that can turn lethal if left untreated. It is found worldwide, whether in endemic zones or as explosive outbreaks in usually unaffected populations (2). Threatening adults as well as children living in developed countries (3), this disease does not spare those traveling to cholera-endemic areas resulting in severe and lethal disease (4). An ideal cholera vaccine would be one offering a single-dose administration, providing fast protection for all age groups and administered to inhabitants of and travelers to countries with increased cholera risk (5).

Cholera originated from its primary source in the 19th century in India, resulting in six subsequent pandemics spread across all continents. Since 2021 and following years of decline, a surge of cholera outbreaks has been observed globally where the case fatality ratios (CFRs) alarmingly surpassed the acceptable levels of <1%. Today’s persistent outbreak represents a resurgence of the ongoing seventh cholera pandemic which started in 1961 (6).

The last major outbreak in Lebanon occurred in the early 1990s, particularly between 1993 and 1994 during the fragile post-war period following the Lebanese Civil War (1975–1990). The war had severely damaged the country’s infrastructure, including vital water treatment and sanitation facilities. The collapse of the health system made it difficult to contain the disease. Thousands of cholera cases were reported during that time, and both morbidity and mortality rates were high, as the health system struggled to cope with the outbreak effectively (7–10).

Lebanon saw a revival of cholera on October 6, 2022, after almost three decades of being cholera-free. The outbreak occurred when the country was already revolving from multiple overlapping crises, including political instability, economic collapse, and the aftermath of the COVID-19 pandemic. These challenges further weakened the public health system and exacerbated longstanding water and sanitation infrastructure vulnerabilities. The outbreak began in Akkar, a region with severe deficiencies in sanitation and water supply, and quickly spread to other regions, including Beirut. By early 2023, thousands of cases had been recorded (10).

Comparing the two outbreaks, the 1990s cholera crisis was primarily driven by the collapse of infrastructure following the civil war, while the 2022 outbreak is occurring amid a severe economic crisis and political dysfunction. The current health system is severely underfunded and ill-prepared for rapid responses to public health emergencies. This time, the international community’s ability to provide swift aid has also been hindered by the economic and political situation, leading to slower and less coordinated containment efforts (7–9).

Both outbreaks disproportionately affected vulnerable populations, but the 2022 outbreak is further complicated by the presence of over 1.5 million Syrian refugees, which has increased the risk of transmission in overcrowded refugee camps that lack adequate sanitation. While public awareness about cholera transmission has improved since the 1990s, systemic issues such as the lack of access to clean water and poor sanitation remain major barriers to effective prevention and control (7–9).

With the declaration of the cholera epidemic in Lebanon, the number of suspected and confirmed cases increased, reaching 7,215 reported cases. Among these, there have been a total of 23 associated deaths, resulting in a case fatality rate of 0.32% (11).

In response, an immediate multi-sectorial work plan among all concerned parties was key to managing the outbreak and limiting the further spread of cases and deaths in the affected areas. It was important to focus on a combination of hygiene, treatment, and preventative measures. These strategies were per the Global Task Force for Cholera Control and essential for reducing endemic and cholera outbreaks (12).

A collaboration between the Lebanese Ministry of Public Health (MoPH) and its partners was initiated to manage and coordinate the cholera response, with the Oral Cholera Vaccine (OCV) immunization campaign being a central component. The MoPH’s Lebanese National Pharmacovigilance Program (LNPVP) was responsible for monitoring and evaluating Adverse Events Following Immunization (AEFIs) with OCVs, ensuring patient and medication safety (13).

In Lebanon, the reporting process for AEFIs adheres to a standardized system aligned with international guidelines. Healthcare providers, vaccine recipients, and caregivers are encouraged to report any suspected adverse events after vaccination. The LNPVP at the MoPH oversees the collection of these reports (14, 15).

AEFI reports can usually be submitted through various channels, including a dedicated hotline, an e-reporting tool, specialized software like the Kobo toolbox directly to the MoPH, or via digital applications like the Medication Safety Application, which simplifies the reporting process by enabling mobile submissions. Serious cases are investigated to determine causality and appropriate response measures (14, 15).

The OCV campaign that was initiated on November 12th, 2022, aimed to control the expansion of cholera in Lebanon, focusing particularly on vulnerable populations (refugees and host communities) in hotspot areas with confirmed cases. The campaign utilized both preventive and reactive vaccination strategies. To optimize this implementation, a hotspot mapping approach was followed to identify the logistics of dose distribution and to achieve the best possible prevention outcomes. Consequently, Lebanese districts were divided into three phases based on a set of criteria relevant to cholera circulation and transmission risk factors (13).

The World Health Organization (WHO) has pre-qualified three oral cholera vaccines (OCVs) for cholera prevention: Dukoral®, Shanchol®, and Euvichol-Plus®. The latter was extensively used during the 2022 vaccination campaigns. While vaccines, like all drugs, typically require registration at the MoPH, Euvichol-Plus® was supplied directly by the WHO due to the urgent need during the cholera outbreak in Lebanon. The immunization campaign was coordinated by MoPH, with WHO providing both technical guidance and vaccine supply, in line with recommendations for use in vulnerable populations and outbreak settings.

During the first cholera outbreak in the 1990s, Dukoral® was the primary vaccine deployed. Dukoral® is an oral, inactivated whole-cell cholera vaccine that includes a component targeting the cholera toxin (CTB), providing dual protection against cholera and *Escherichia coli*-induced diarrhea. As one of the earliest cholera vaccines developed, it played a significant role in initial outbreak responses and became more widely used during that time (1, 16).

Shanchol® developed later, gained prominence in cholera outbreaks from the 2000s onwards. Like Dukoral, Shanchol is an oral, killed whole-cell vaccine, but it lacks the cholera toxin B subunit (CTB). It became more widely used due to its effectiveness and suitability for mass vaccination campaigns in endemic areas (1, 17).

In the 1990s, however, vaccines were not as widely deployed for mass immunization during cholera outbreaks as they are today. Public health strategies at the time focused more on improving water, sanitation, and hygiene (WASH) measures, along with oral rehydration therapy (ORT), to manage and prevent cholera transmission (1).

Euvichol-Plus® is used for active immunization to protect against cholera. Administered orally, it is a liquid formulation of OCV containing inactivated *V. cholerae* strains. It presents a good safety profile, with a list of relatively non-serious AEFIs including fever, abdominal pain, diarrhea, nausea/vomiting, headache, and myalgia (18).

Euvichol-Plus was developed by EuBiologics. It has been utilized in various countries as part of cholera outbreak responses and preventive campaigns. Bangladesh was one of the earliest adopters, deploying Euvichol-Plus during mass vaccination campaigns in 2016 in high-risk areas like Dhaka, as part of a broader initiative to control cholera in endemic zones. Similarly, in Haiti, Euvichol-Plus was used following the devastating 2016 Hurricane Matthew, which led to a resurgence of cholera. The Democratic Republic of the Congo (DRC) incorporated the vaccine into its emergency response to cholera outbreaks in 2017, targeting vulnerable populations in conflict-affected regions. Yemen, amid its ongoing humanitarian crisis, introduced Euvichol-Plus in 2018 to limit its severe cholera epidemic, one of the largest globally at that time. Additionally, Mozambique deployed Euvichol-Plus in 2019 following the impact of Cyclone Idai, which exacerbated cholera transmission due to flooding and poor sanitation. More recently, countries like Nigeria (2021), Malawi (2022), and Lebanon (2022) have utilized Euvichol-Plus during their outbreaks, as part of national cholera control efforts in collaboration with the World Health Organization (WHO) and other international health partners (1, 19–21).

The current study’s main objective is to describe and analyze AEFIs temporally associated with the Euvichol-Plus®, following the deployment of the national OCV campaign from November 12th, 2022 to February 19th, 2023, in Lebanon. This study covers adverse events reports received at the LNPVP’s passive surveillance system, for the vaccination campaign’s Phases I, II, and III. As the LNPVP received reports of adverse events related to Euvichol-Plus® which was the primary OCV administered during the three Campaign Phases, this study will mainly focus on related OCV AEFIs.

To the best of our knowledge, and based on literature review, this study is the first national and regional effort to present real-world data of AEFIs associated with the OCV Euvichol-Plus®. It provides a valuable reference for the widespread use of OCVs in developing countries, supporting cholera eradication efforts.

## 2 Methods

### 2.1 AEFI reporting system

The Lebanese National Pharmacovigilance Program (LNPVP) established a procedure to manage reported adverse events following the Euvichol-Plus® vaccine. AEFIs were reported through one of the following means: the OCV Vaccine Hotline Call Center (1787) (22), the LNPVP Landline (23), or the Kobo toolbox, a Software created by the MoPH to report AEFIs by Health Care Professionals (HCPs) (24). During the immunization campaign in Lebanese districts, vaccine administrators informed recipients about the procedure for reporting AEFI related to OCV. This information was communicated directly during vaccine administration and detailed in flyers distributed to all recipients. The hotline number (1787) was also available to address any queries or uncertainties regarding AEFI, ensuring recipients had access to support and information.

The reporting tools collected essential details, including the reporter’s name, contact information, age, gender, administered vaccine, and the adverse event(s) temporarily associated with the administered vaccine. Case reports were screened and validated for data completion. Direct follow-up with the initial reporter was carried out for incomplete or inconsistent case reports.

### 2.2 Study design

This is a retrospective observational study. It includes AEFIs with OCV Euvichol-Plus®, received by the LNPVP through passive surveillance from November 2022 to February 2023. During the study period, 1,500,000 doses of the Euvichol-Plus® vaccine were administered, reaching 621,382 individuals, with 46 cases of AEFIs reported. Additionally, 149,671 people were engaged through community outreach and door-to-door campaigns to raise awareness about Cholera prevention and treatment in high-risk and vulnerable areas. Meanwhile, 33 healthcare professionals from 13 public and private hospitals received training to manage Cholera cases in children (25).

### 2.3 Classification of reports

Case reports were classified as either serious or non-serious. According to the WHO definition of seriousness criteria, a serious case report includes AEFIs that result in death, hospitalization, or prolongation of an existing hospitalization, persistent or significant disability or incapacity, congenital anomaly/birth, defect or is life-threatening (26).

Concerning serious case reports, the Pharmacovigilance (PV) team followed up with the reporter and his physician or family members. The cases were investigated using the WHO AEFI investigation form (27), and causality assessment was conducted using the WHO AEFI Causality Assessment Software (28).

All case reports were entered into VigiFlow®, the national web-based Individual Case Safety Report (ICSR) data management system. The software supports the collection, processing, analysis, and sharing of Adverse Drug Reaction (ADR) and AEFI reports (29). Data entry was done using standardized terms through the Medical Dictionary for Regulatory Activities (MedDRA®) (30).

As for the age, VigiBase® the WHO global database system, divides patients’ age into the following ranges: 0–1 year, 2–11 years, 12–17 years, 18–44 years, 45–64 years, 65–74 years.

### 2.4 Data extraction

Case reports were extracted from VigiLyze®, which is a World Health Organization-Uppsala Monitoring Center (WHO-UMC) signal detection and signal management tool (31). The number of administered vaccine doses was obtained from MoPH’s official website (32).

### 2.5 Statistical analysis

Descriptive analysis was performed, and results were reported as counts and percentages. In addition, the proportion of ICSRs reported among different age categories and gender were computed. For the age, mean and standard deviation were also computed. Statistical analysis was performed using the SPSS software (version 23.0). Categorical variables were compared using Pearson’s *χ*^2^ test. Statistical significance was set at *p* < 0.05.

## 3 Results

### 3.1 Description of case reports following Euvichol-Plus® vaccine

Table 1 represents case reports following Euvichol-Plus® vaccine administration. From November 12th, 2022 to February 19th, 2023, the LNPVP received a total of 46 case reports, corresponding to 115 AEFIs with Euvichol-Plus®. Most of the reported cases were non-serious (82.6%), with females (56.5%), and reporters aged between 2 years old and 11 years old (41.3%) contributing to the highest proportion of ICSRs received. The age range of the reporters was (1–74) years old. Most of the cases were assigned the origin of Beqaa governorate (39.1%) and reported using the hotline call center (67.4%) (Table 1).

**Table 1:** Description of and case reports following Euvichol-Plus® vaccine.

Case reports	*N* = 46 (%)
Serious case reports	8 (17.4)
Non-serious case reports	38 (82.6)
Gender	
Female	26 (56.5)
Male	20 (43.5)
Age groups (years)	
0–1	1 (2.2)
2–11	19 (41.3)
12–17	5 (10.9)
18–44	14 (30.4)
45–64	4 (8.7)
65–74	3 (6.5)
Age range	(1–74) years-old
Governorate	
Baalbeck-Hermel*	14 (30.5)
Bekaa**	18 (39.1)
North***	8 (17.4)
Akkar	4 (8.7)
Mount Lebanon****	2 (4.3)
Means of reporting	
1787 Hotline call center	31 (67.4)
Landline	11 (23.9)
KoboToolbox AEFIs: AEFIs software for reporting	4 (8.7)

*Baalbeck-Hermel: Baalbeck.

**Beqaa: Zahle, and west beqaa.

***North: Tripoli, El Minieh, Zgharta.

****Mount Lebanon: El Metn, and Baabda.

### 3.2 Reported AEFI with Euvichol-Plus® vaccine and their correlation with age and gender

The AEFIs with Euvichol-Plus® vaccine are reported systematically in Table 2, with each number of reports corresponding to the specific adverse event reported. The top 3 reported AEFIs were fever (39.13%), diarrhea (30.43%), and vomiting (30.43%).

**Table 2:** Reported adverse events following Euvichol-Plus® vaccine.

Adverse events following immunazation (AEFI)	Number of reports *N* = 46 (%)
Fever	18 (39.13)
Diarrhea	14 (30.43)
Vomiting	13 (28.26)
Abdominal pain	13 (28.26)
Fatigue	7 (15.22)
Headache	7 (15.22)
Weakness	4 (8.70)
Nausea	4 (8.70)
Dyspnea	4 (8.70)
Overdose	3 (6.52)
Vertigo	3 (6.52)
Epigastric pain	2 (4.35)
Loss of taste	2 (4.35)
Chills	2 (4.35)
Dizziness	2 (4.35)
Allergic reaction	2 (4.35)
Myalgia	2 (4.35)
Angioedema	1 (2.17)
Loss of smell	1 (2.17)
Burning sensation	1 (2.17)
Chest pain	1 (2.17)
Dehydration	1 (2.17)
Bitter taste	1 (2.17)
Hyperglycemia	1 (2.17)
Influenza like illness	1 (2.17)
Paranesthesia of limbs	1 (2.17)
Rash	1 (2.17)
Runny nose	1 (2.17)
Syncope	1 (2.17)
Tachycardia	1 (2.17)
Total AEFI	**115**

The bold number reflects the total number of reported AEFI with OCV for the above-listed case reports.

By correlating these AEFIs with age and gender, fever was significantly reported among vaccine recipients between 2 and 11 years old (*p* < 0.05). As for headache, it was more common among females than males (26.9% vs. 0%, *p* < 0.05), and in those between 12 and 17 years old (*p* < 0.05; Table 3).

**Table 3:** Reported Euvichol-Plus® vaccine adverse events and their correlation with age and gender.

	Gender		*p* value	Age categories (years)						*p* value
	Female *N* = 26 (%)	Male *N* = 20 (%)		0–1 *N* = 1 (%)	2–11 *N* = 19 (%)	12–17 *N* = 5 (%)	18–44 *N* = 14 (%)	45–64 *N* = 4 (%)	65–74 *N* = 3 (%)	
Fever	9 (34.6)	9 (45)	0.474	0	13 (68.4)	2 (40)	2 (14.3)	0	1 (33.3)	0.006*
Diarrhea	8 (30.8)	6 (30)	0.955	1 (100)	6 (31.6)	3 (60)	2 (14.3)	0	2 (66.7)	0.065
Vomiting	8 (30.8)	5 (25)	0.667	1 (100)	5 (26.3)	3 (60)	3 (21.4)	1 (25)	0	0.286
Abdominal pain	8 (30.8)	5 (25)	0.667	0	4 (21.1)	1 (20)	6 (42.9)	2 (50)	0	0.552
Fatigue	3 (11.5)	4 (20)	0.682	0	4 (21.1)	0	1 (7.1)	1 (25)	1 (33.3)	0.517
Headache	7 (26.9)	0	0.003*	0	0	2 (40)	5 (35.7)	0	0	0.02*
Weakness	3 (11.5)	1 (5)	0.622	0	4 (21.1)	0	0	0	0	0.389
Nausea	3 (11.5)	1 (5)	0.622	0	3 (15.8)	0	1 (7.1)	0	0	0.905
Dyspnea	2 (7.7)	2 (1)	1	0	1 (5.3)	0	1 (7.1)	1 (25)	1 (33.3)	0.451
Vertigo	3 (11.5)	0	0.058	0	0	2 (40)	1 (7.1)	0	0	0.08
Overdose	1 (3.8)	2 (10)	0.572	0	2 (10.5)	1 (20)	0	0	0	0.507
Epigastric pain	1 (3.8)	1 (5)	1	0	0	1 (20)	1 (7.1)	0	0	0.325
Loss of taste	2 (7.7)	0	0.498	0	0	0	2 (14.3)	0	0	0.486
Allergic reaction	1 (3.8)	1 (5)	1	0	0	0	1 (7.1)	1 (25)	0	0.202
Chills	2 (7.7)	0	0.498	0	0	0	2 (14.3)	0	0	0.486
Dizziness	1 (3.8)	1 (5)	1	0	1 (5.3)	0	1 (7.1)	0	0	1
Myalgia	2 (7.7)	0	0.498	0	0	0	2 (14.3)	0	0	0.486
Angioedema	1 (3.8)	0	1	0	0	0	1 (7.1)	0	0	0.587
Loss of smell	1 (3.8)	0	1	0	0	0	1 (7.1)	0	0	0.587
Burning sensation	1 (3.8)	0	1	0	0	0	1 (7.1)	0	0	0.587
Chest pain	1 (3.8)	0	1	0	1 (5.3)	0	0	0	0	1
Dehydration	0	1 (5.0)	0.435	0	0	1 (20.0)	0	0	0	0.283
Flu like symptoms	0	1 (5.0)	0.435	0	0	0	1 (7.1)	0	0	0.587
Syncope	0	1 (5.0)	0.435	0	0	0	0	0	1 (33.3)	0.087
Bitter taste	1 (3.8)	0	1	0	0	0	1 (7.1)	0	0	0.587
Tachycardia	0	1 (5.0)	0.435	0	0	0	1 (7.1)	0	0	0.587
Paresthesia of limbs	0	1 (5.0)	0.435	0	0	0	1 (7.1)	0	0	0.587
Runny nose	1 (3.8)	0	1	0	0	0	1 (7.1)	0	0	0.587
Rash	1 (3.8)	0	1	0	0	0	1 (7.1)	0	0	0.587
Hyperglycemia	0	1 (5.0)	0.435	0	0	0	0	0	1 (33.3)	0.087

Results are presented as frequency (*N*) and percentage (%).

*p < 0.05.

The bold values are the significant ones.

### 3.3 Reported AEFI with Euvichol-Plus® vaccine and their time to onset

Table 4 represents the top 15 reported reactions and their distribution, according to the time of onset after vaccination. Most reactions were reported 1 day following vaccination (31 reactions) followed by day zero (same day of vaccination) following vaccination (23 reactions).

**Table 4:** Reported adverse events following immunization with Euvichol-Plus® vaccine.

Time to onset	Day 0	Day 1	Day 2	Day 3	Day 5	Unknown
Fever	4 (17.5)	9 (29.2)	0	0	3 (20)	2 (25)
Diarrhea	6 (26.0)	4 (12.9)	1 (6.25)	0	3 (20)	0
Vomiting	3 (13.0)	6 (19.4)	1 (6.25)	0	3 (20)	0
Abdominal pain	3 (13.0)	1 (3.2)	7 (43.75)	0	0	2 (25)
Fatigue	0	3 (9.6)	2 (12.5)	0	0	2 (25)
Headache	0	1 (3.2)	2 (12.5)	1 (20)	3 (20)	0
Weakness	0	4 (12.9)	0	0	0	0
Nausea	0	0	1 (6.25)	1 (20)	0	2 (25)
Dyspnea	2 (8.7)	1 (3.2)	0	1 (20)	0	0
Overdose	2 (8.7)	1 (3.2)	0	0	0	0
Vertigo	0	0	0	0	3 (20)	0
Epigastric pain	0	1 (3.2)	1 (6.25)	0	0	0
Loss of taste	0	0	1 (6.25)	1 (20)	0	0
Allergic reaction	2 (8.7)	0	0	0	0	0
Dizziness	1 (4.4)	0	0	1 (20)	0	0
Total	23	31	16	5	15	8

### 3.4 Description of the serious case reports following Euvichol-Plus® vaccine

Table 5 represents eight serious cases received by the LNPVP in the identified study period. They were equally divided between females and males. Reporters’ mean age was 32.25 ± 23.536. All serious cases required Emergency Room (ER) visits (Table 5).

**Table 5:** Serious case reports following Euvichol-Plus® vaccine.

Serious case		Case 1	Case 2	Case 3	Case 4	Case 5	Case 6	Case 7	Case 8
Patient details	Gender	Female	Male	Male	Male	Male	Female	Female	Female
	Age (years)	39	14	74	60	14	13	16	28
	Mean age ± SD	32.25 ± 23.536							
	Underlying condition	None	None	Dyslipidemia Hypertension Myocardial Infarction	Diabetes (HbA1 = 5.9%). Iron deficiency anemia. Allergies to medications and food (penicillin, cephalosporin, goat cheese, shrimps, and others)	None	None	None	None
AEFI details	AEFI	Allergic reaction	Vomiting Epigastric Pain Chills	Severe Diarrhea	Allergic reaction	Overdose Watery diarrhea Vomiting Dehydration	Fever Vomiting Severe Diarrhea Vertigo Headache	Fever Vomiting Severe Diarrhea Vertigo Headache	Fever Vomiting Severe Diarrhea Vertigo Headache
	The time interval between the vaccine & AEFI	5 h	1 day	3 h	1 h	1 day	5 days	5 days	5 days
	Seriousness	Serious – ER Visit	Serious – ER Visit	Serious – ER Visit	Serious – ER Visit	Serious – ER visit	Serious – ER Visit	Serious – ER Visit	Serious – ER Visit
	Causality Assessment	Consistent	Consistent	Consistent	Consistent	Consistent	Indeterminate	Indeterminate	Indeterminate

## 4 Discussion

Under-reporting during the national cholera vaccination campaign in Lebanon was highlighted by the rather low AEFI reporting rate of 0.101 AEFIs per 1,000 doses.

This study reports a significant effort in presenting and analyzing AEFI associated with the OCV Euvichol-Plus®. These findings are instrumental in addressing the gap in vaccine safety, providing a reference point for AEFI monitoring in forthcoming cholera vaccination campaigns. Moreover, this study identified a novel serious AEFI that has not been previously associated with OCVs. Thereby adding to available OCVs literature, which holds noteworthy public health implications.

Starting with this study’s reporting rate is lower than the 5–10 AEFIs (per 1,000 doses) reported during two pilot OCV campaigns in Haiti. These campaigns included Shanchol OCV (33). Similarly, it is significantly lower than the 11.3 AEFIs (per 1,000 doses) of COVID-19 vaccines reported during the first 4 months after initiation of the national Lebanese COVID-19 vaccination campaign (15). Although not as cost-effective as the COVID-19 vaccination campaign, the focus of the OCV deployment strategy was to prioritize hotspots harboring risk factors for transmission, to completely eradicate the disease. The door-to-door vaccination approach was successful in previous cholera vaccination campaigns (34). However, multiple implementing partners were involved in the rollout of the OCV campaign, which limited the effort of addressing the significance of AEFI reporting. This has possibly led to a disjointed AEFI reporting approach, unlike the COVID-19 vaccination campaign where the MOPH’s PV team disseminated targeted training (35) on available reporting means and the importance of reporting in general (36).

AEFIs reported in this study were classified in the majority as non-serious. Results are confirmed by a 5-year meta-analysis on global OCV use conducted by the Global Task Force on Cholera Control (37). A study published by Bwire et al. in Uganda reports very few AEFIs, as in this study (38). Their results are per previous OCV campaigns outside Uganda, with scarce AEFI reports (39). Adverse events were reported mostly as mild or moderate while being self-limited (38).

Fever (39.1%) was the most reported AEFI, with a significant association with the vaccine in the 2–11 years old age group (*p* = 0.006). This result is similar to previously published studies, involving Shanchol OCV in Bangladesh (33) and pediatric vaccines in India, in general (40).

Noting that Euvichol-Plus®‘s oral administration excludes AEFIs linked to injection-site reactions, which are the second most reported AEFI in most studies (41). Instead, diarrhea (30.4%) and vomiting as well as abdominal pain (28.3% each) were expectedly observed. It is in sync with the vaccine’s safety profile, suggesting a plausible incidence of gastrointestinal symptoms (42).

Hence, AEFIs with the highest prevalence are found in the following published studies (33), such as the one reporting AEFIs following OCV administration in Haiti. Haiti’s most reported adverse events were similar to the ones reported in this study where they included nausea, vertigo, and abdominal pain (33). In addition, they did not report any major adverse events (33), which confirms our findings throughout LMICs. Another study confirms that common symptoms reported following OCV vaccination are abdominal pain, diarrhea, fever, nausea, and headaches (38), thereby verifying the main AEFIs reported in this study.

This descriptive analysis reported two main factors; gender and age-category, correlated to reported AEFIs with Euvichol-Plus® OCV. First, reported AEFIs were noted at a higher percentage in females (56.5%), with a significant association between female gender and headaches specifically (*p* = 0.003). This gender-based immunological response is well documented in the literature, as reported by Klein et al. Their review suggests that females have consistently reported more frequent and severe reactions to measles, human papillomavirus, influenza, hepatitis B, yellow fever, pneumococcal, and shingles vaccines. Reflecting on the possibility of either a greater innate, humoral, and cell-mediated inflammatory response among females (when compared to males), or on a reporting bias (14, 43). Whitaker et al. further explain this differential immune response to vaccines as not solely based on sex hormones, but also on the more vigorous response of females to certain vaccines (more elevated IgG titers), which makes them require lower doses than their male counterparts (44).

The second factor correlated to reported AEFIs is age category, whereby the highest prevalence of AEFIs following the OCV Euvichol-Plus® was reported among children aged between 2 and 11 years old (41.3%). These results are comparable to those of active surveillance of AEFIs in Uganda, following a Measles & Rubella vaccine (MR) and Bivalent Oral Polio vaccine (bOPV) immunization campaign. 62.8% of the reported AEFIs were observed in children between 5 and 15 years of age (45). A plausible explanation is that children are usually more prone to developing infections and inflammations, given their immune system’s vulnerability, which leads to a greater risk of adverse events. One patient stood out from the usual serious AEFIs reports, reporting persistent angioedema and rash, symptomatic of an allergic reaction. This notable observation was made, based on the fact that all serious AEFIs reported in this study were flagged in Euvichol-Plus® safety profile (18). This study’s team considered the allergic reaction AEFI to be noteworthy, due to its infrequent occurrence following OCVs and its rare documentation in the contextual literature. Based on our knowledge, angioedema is not identified as a common or rare side effect of Euvichol-Plus® in either clinical trials or post-marketing surveillance. However, it is established that any vaccine has the potential to elicit an allergic reaction, even in individuals without previous allergic episodes (46). In this case, one or more components of the vaccine could act as the offending agent. Following vaccine exposure, the immune system may mount an IgE-mediated response to vaccine components, triggering the release of inflammatory mediators. It causes vasodilation, increased vascular permeability, and tissue edema, ultimately resulting in the manifestation of angioedema (46).

After computing the reasonable temporal relationship between AEFI and vaccine intake, biological plausibility of the event, absence of other causal factors and the patient’s medical history, the association of angioedema with Euvichol-Plus® was assessed as ‘consistent’ by both the PV team and the Serious AEFI Special Committee (42). Therefore, this allergic reaction is worth documenting, to raise awareness targeted to vaccine recipients and healthcare providers during upcoming cholera immunization campaigns.

In addition to raising awareness toward reporting AEFIs for cholera vaccines, efficient cholera prevention and control are through offering safe water, proper hygiene and improving sanitation. WHO further advises that OCV campaigns must be complemented by these traditional control measures for cholera (47), including WASH interventions to offer a constructive approach to controlling and preventing cholera (33).

## 5 Limitations

The AEFIs reported in the present study are received by the LNPVP through a passive surveillance system which entails several limitations. Inherent disadvantages include under-reporting and incompleteness of the received AEFI reports. Additionally, due to the limited study sample size and timeframe of the vaccination campaign, an underestimation of the true incidence of AEFI with the OCV Euvichol-Plus® is plausible. Moreover, adults are vaccinated less frequently than children for OCV, which limits further the ability to identify AEFI in adult populations. It is also plausible that some AEFI cases were missed by healthcare professionals, who are in charge of reporting AEFIs via the Kobo Toolbox.

Moreover, the results of the study may have limited generalizability, as the OCV vaccination focused specifically on vulnerable populations, such as refugees and host communities. These groups often have different health conditions, living environments, and access to healthcare compared to the general population, which could influence both the vaccine’s effectiveness and the occurrence of adverse events. Therefore, while the findings provide valuable understandings of these specific populations, caution should be exercised when applying the conclusions to the broader population, as the unique characteristics of the study group may not reflect those of the general public.

Despite the listed limitations, it is believed that they do not significantly affect or invalidate the study’s results or the reliability of its conclusions, as the key findings remain supported by the data and consistent with existing literature.

## 6 Conclusion

The LNPVP, under the MOPH, is actively advancing the monitoring of vaccines and medications, with a particular focus on strengthening the surveillance of AEFI in Lebanon. This study provides significant insights into the safety profile of the OCV Euvichol-Plus®, emphasizing the need for further research into hypersensitivity reactions and safety assessments across more diverse population groups.

The findings reveal that most adverse events were non-serious, with children and females identified as key groups experiencing distinct reactions, such as fever in younger children and headaches more commonly reported among females. These observations are consistent with broader vaccine studies, suggesting that females and certain age groups may have more pronounced immune responses, leading to more frequent adverse events. However, the occurrence of serious cases, although rare, highlights the necessity for ongoing vigilance and active AEFI monitoring.

To enhance vaccine safety in low- to middle-income countries (LMICs), establishing baseline AEFI rates is crucial. The current lack of expected AEFI data in LMICs presents challenges for healthcare professionals and policymakers in developing evidence-based vaccination strategies. Reliable data will enable more informed decision-making for future vaccination campaigns, ensuring better preparedness and improved public health outcomes in Lebanon.

## Data Availability

The datasets presented in this study can be found in online repositories. The names of the repository/repositories and accession number(s) can be found in the article/supplementary material.
